# The LINC00452/miR-204/CHST4 Axis Regulating Thymic Tregs Might Be Involved in the Progression of Thymoma-Associated Myasthenia Gravis

**DOI:** 10.3389/fneur.2022.828970

**Published:** 2022-03-30

**Authors:** Fuqiang Wang, Hanlu Zhang, Guanghao Qiu, Zhiyang Li, Yun Wang

**Affiliations:** ^1^Department of Thoracic Surgery, West China Hospital, Sichuan University, Chengdu, China; ^2^West China School of Medicine, Sichuan University, Chengdu, China

**Keywords:** thymoma, myasthenia gravis, ceRNA network, Treg, tumor infiltrating immune cells

## Abstract

**Background:**

Myasthenia gravis (MG) is an autoimmune disease that mainly affects neuromuscular junctions and is usually associated with immune disorders in the thymoma. The competitive endogenous RNA (ceRNA) hypothesis has been demonstrated to be an intrinsic mechanism regulating the development of several autoimmune diseases; however, the mechanism where the ceRNA network regulates immune cells in patients with thymoma-associated MG (TAMG) has rarely been explored.

**Methods:**

RNA-seq data and clinical information of 124 patients with thymoma were obtained from The Cancer Genome Atlas (TCGA) database. The patients were divided into two groups according to whether they were diagnosed with MG. We applied the propensity score matching method to reduce the incidence of baseline confounders. We then constructed a ceRNA network with differentially expressed RNAs between the groups based on four public databases. The expression of genes of interest was validated by qPCR. Moreover, we predicted the immune cells that infiltrated the thymoma and then analyzed the association between immune cells and RNA in the ceRNA network. To further determine the function of the mRNAs associated with immune cells in patients with TAMG, we performed gene set enrichment analysis in thymoma patients with MG.

**Results:**

After matching, 94 patients were included in the following analysis. A total of 847 mRNAs, 409 lncRNAs, and 45 miRNAs were differentially expressed between the groups. The ceRNA network, including 18 lncRNAs, four miRNAs, and 13 mRNAs, was then constructed. We then confirmed that CHST4 and LINC00452, miR-204-3p and miR-204-5p were differentially expressed between patients with TAMG and thymoma patients without MG (NMG) by qPCR. Moreover, we found that the percentage of predicted regulatory T (Treg) cells was significantly decreased in patients with TAMG. Further analysis indicated that the LINC00452/miR-204/CHST4 axis might regulate thymic regulatory T cells (Tregs) in the progression of MG.

**Conclusions:**

In this research, we constructed a ceRNA network involved in the progression of TAMG, discovered that thymic Tregs were significantly decreased in patients with TAMG, and assumed that the LINC00452/miR-204/CHST4 axis may regulate thymic Tregs in the development of TAMG. These findings may deepen our understanding of the roles of the ceRNA network in regulating TAMG and highlight the function of CHST4 in recruiting peripheral T cells in the progression of TAMG.

## Introduction

Myasthenia gravis (MG) is an autoimmune disease that mainly affects the neuromuscular junction of patients (1, 2). In most cases, aberrantly elevated antibodies targeting the acetylcholine receptor (AChR) can lead to a disorder of the neuromuscular junction (2). Furthermore, AChR-MG is usually associated with morphological and functional changes in the thymus, especially thymoma. It has been reported that more than 10% of AChR-MG patients are affected by thymoma (2, 3); hence, thymoma-associated MG (TAMG) is classified as one of the major subgroups of MG (in clinical terms), and thymectomy is strongly recommended to manage TAMG (2, 3).

Previous work has shown that the pathological changes in thymoma play crucial roles in both the occurrence and progression of TAMG (2, 3). In thymoma, due to the deficiency of thymic medulla, the negative selection of thymocytes is impaired so that the abnormal T cells with autoimmune potential may not be eliminated (3–5). Meanwhile, other research shows that non-coding RNAs participate widely in immune dysfunction in TAMG (5–9). Li et al. (6) found that miR-125a-5p was abnormally elevated in the thymus of TAMG, which could target Foxp3 and lead to a decrease in Treg cells. In addition, the elevation of miR-19b-5p was detected in MG-related thymoma, which could target thymic stromal lymphopoietin, resulting in the imbalance of T cells (7).

Salmena presented a competitive endogenous RNA (ceRNA) hypothesis which proposed that messenger RNA (mRNA) can crosstalk with noncoding RNA by competitive binding to the same microRNA through common microRNA response elements (10, 11). In recent years, various researchers have confirmed that this regulatory mechanism exists widely in the pathogenesis of several autoimmune diseases, including Systemic lupus erythematosus (SLE) (12), rheumatoid arthritis (13), and MG. Wang and colleagues found that SNHG16 could compete with interleukin-10 by binding to let-7c-5p in peripheral blood mononuclear cells (PBMCs) of patients with MG (14, 15). The elevated SNHG16 in the peripheral blood of patients with MG increased the expression of IL-10, which promoted B-cell activation and was associated with anti-AChR antibody production in juvenile MG (14). Despite the recognized importance of the ceRNA network in the pathogenesis of the peripheral immune system in MG, its role in the pathological process of the thymus remains to be fully explored.

Therefore, this research was conducted to establish a ceRNA network in TAMG thymoma to further improve our understanding of the mechanisms of immune dysfunction in TAMG. By using the data pertaining to patients with thymoma from TCGA database, we constructed the ceRNA network based on the differentially expressed genes, predicted, and compared the tumor-infiltrating immune cells (TIICs) between thymoma and patients with TAMG, and explored the possible mechanisms whereby the ceRNA network regulated TIICs in TAMG.

## Materials and Methods

### RNA-Seq Data Acquisition

The RNA expression profiles and clinical data of patients thymoma were obtained from The Cancer Genome Atlas (TCGA) database. We downloaded the clinical data of 124 patients, the RNA-seq data of 121 samples, and the miRNA-isoform-seq data of 126 samples in TCGA–THYM datasets for further processing. The sequencing data of normal thymus and patients lacking a history of MG were excluded from the downstream analysis. According to the history of MG, we divided patients into a thymoma-associated MG (TAMG) group and thymoma patients without MG (NMG) group.

### Propensity Score Matching (PSM)

Propensity score matching was performed to control for baseline confounders. Thereafter, the sequencing data belonging to matched patients were selected and subjected to the following data-processing step: the R package “MatchiIt” was used to conduct PSM (16). Propensity matching was performed in a 1:5 ratio (TAMG vs. NMG) using the nearest neighbor method within a distance of 0.2. The factors potentially associated with MG, including age, sex, WHO histological type, and Masaoka stage, were matched between the groups (17, 18).

Demographic information was compared between the groups before and after matching. The counting data were expressed as a ratio and analyzed by the chi-squared test or Fisher's exact test. The measurement data are shown as median points and were studied by Student's *t* test. A *p*-value ≤ 0.05 was deemed statistically significant.

### RNA-Seq Data Processing

First, the genes expressed in fewer than 50% of samples were removed. Next, the ENSEML ID for protein-coding RNA and lncRNA was annotated according to GENCODE Release 22. The following transcript biotypes were defined as lncRNAs: 3prime overlapping ncRNA, antisense, bidirectional promoter lncRNA, lincRNA, macro lncRNA, non-coding, processed transcript, sense intronic, and sense overlapping. Thereafter, the fragments per kilobase million values of the remaining genes were transformed into transcripts per kilobase million (TPM) values.

### Differential Gene Expression Analysis

Differential gene expression analysis was conducted using the R package “DEseq2” to identify the differentially expressed mRNAs (DEmRNAs), lncRNAs (DElncRNAs), and miRNAs (DEmiRNAs) (19). The *p*-value was corrected for the false discovery rate (FDR) using the Benjamini–Hochberg method. The threshold for differential expression was set as FDR < 0.05 and |log_2_ Fold Change (FC)| > 1.

### Functional and Pathway Enrichment Analysis

Gene Ontology (GO) functional enrichment and Kyoto Encyclopedia of Genes and Genomes (KEGG) pathway enrichment analyses of differentially expressed RNAs were performed using the R package “clusterprofiler” (20–23). The *p*-value was corrected by the Benjamini–Hochberg method (adj.p). The functional categories and pathways with adjusted *p*-values < 0.05 were deemed to have been significantly enriched.

### Construction of the ceRNA Network

First, based on the miRcode database, we predicted DElncRNA-DEmiRNA pairs that may interact with each other (24). Then, according to the DEmiRNAs in the predicted DElncRNA–DEmiRNA pairs, we predicted the DEmRNA–DEmiRNA pairs using the TargetScan, miRTarBase, and miRDB databases (25–27). Only the DEmRNA–DEmiRNA pairs that were successfully predicted in all three databases were selected to build the network. Thereafter, based on the predicted DElncRNA–DEmiRNA pairs and DEmRNA–DEmiRNA pairs, we established the ceRNA network and visualized the network with the R package “ggalluvial.”

### The Immune Features of Thymoma

We used the “Estimation of STromal and Immune cells in MAlignant Tumors using Expression data” (ESTIMATE) algorithm to calculate and compare the ESTIMATE score, tumor purity score, stromal score, and immune score between the groups to analyze the tumor microenvironment of thymoma (28). Then, we evaluated the TIICs in thymoma with the “cell-type identification by estimating relative subsets of RNA transcripts” (CIBERSORT) algorithm in patients with TAMG (29). The results were visualized with the R packages “ggpubr” and “ggplot2.”

### Investigation of the Association Between TIICs and DEmRNAs and the Correlation Between DEmRNAs and DElncRNAs in the ceRNA Network

Based on the fraction of TIICs calculated by CIBERSORT and the TPM of DEmRNAs in the ceRNA network, we assessed the correlation between TIICs and DEmRNAs using the Pearson correlation coefficient (*r*) with the R package “corrplot.” Additionally, we calculated the correlation coefficients between DElncRNAs and DEmRNAs in the ceRNA network by the Pearson correlation coefficient (*r*). Values of *p* < 0.05 and |*r*| ≥ 0.3 were considered indicative of factors that were significantly correlated with each other; 0.3 ≤ |*r*| < 0.5 was indicative of a weak correlation, 0.5 ≤ |*r*| < 0.7 was indicative of a moderate correlation, 0.7 ≤ |*r*| < 0.9 was indicative of a high correlation, and 0.9 ≤ |*r*| < 0.1 was indicative of a very high correlation.

### Functional Prediction of Selected DEmRNAs

According to the median TPM of selected DEmRNAs, we divided patients with TAMG into high-expression and low-expression groups. Next, we performed gene set enrichment analysis (GSEA) between the high- and low-expression groups using the R package “clusterprofiler” to evaluate the function of this mRNA in the development of TAMG (20, 30). The functional categories and pathways with *p*-value < 0.05 and FDR-corrected *p*-value (*q*-value) < 0.25 were deemed to be significantly enriched.

### Tissue Collection, RNA Extraction, and Quantitative PCR

The thymic tissues for qPCR were taken from 5 patients with TAMG and 4 patients with thymoma who underwent thymectomy in our center. The study was approved by the Ethics Committee of West China Hospital of Sichuan University (2021-578). A written informed consent was obtained from all the patients.

Total RNA was extracted with TRIzol reagent (Ambion), separated with chloroform, precipitated with isopropyl alcohol, washed with 75% ethanol, and redissolved in water. cDNA was synthesized using PrimeScript™ RT reagent Kit with gDNA Eraser (TAKARA). Stem-poop RT primers were used for the reverse transcription of miR-204-3p and miR-204-5p. ACTINB and U6 were used as reference genes for mRNA (lncRNA) and miRNA, respectively, to normalize the expression of the genes of interest. qPCR was performed by using TB Green^®^ Premix Ex Taq™ II (TAKARA). The PCR conditions were as follows: 95°C for 30 s, followed by 40 cycles of 95°C for 30 s and 60°C for 30 s. Melting curve analyses indicated primer specificities. The primer sequences are detailed in **Table 2**.

## Results

### Demographic Data

The demographic data of 124 patients were obtained from the TCGA database. Eight patients were excluded for lacking a history of MG (three patients) and RNA-seq data (five patients). Meanwhile, according to the definition of thymoma in the 2015 World Health Organization (WHO) classification of tumors of the thymus, eleven patients with type C thymoma (thymic carcinoma) were excluded (17). Thus, the demographic data of a total of 105 patients were analyzed.

The demographic data are summarized in Table 1. Before matching, the histological type of thymoma was significantly different between the groups (*p* = 0.025). To reduce the effect of baseline confounders, we then performed PSM: 94 patients were successfully matched, including 27 patients in the TAMG group and 67 patients in the NMG group. After matching, there was no significant difference in baseline characteristics between the groups.

**Table 1:** Demographic information before and after propensity score matching.

**Patient's characteristic**		**Before matching**			**After matching**		
		**TAMG (*N* = 34)**	**NMG (*N* = 71)**	***P*-value**	**TAMG (*N* = 27)**	**NMG (*N* = 67)**	***P*-value**
Gender				0.835			0.624
	Male	17 (50.00%)	38 (53.52%)		13 (48.15%)	36 (53.73%)	
	Female	17 (50.00%)	33 (46.48%)		14	31	
Age, y, median age (range)		55.5 (17–79)	60 (31–84)	0.259	59 (17–79)	61 (31–84)	0.218
BMI, Kg/m^2^, median BMI (range)		26.64 (18.08–50.45)	26.67 (17.76–50.61)	0.835	27.11 (18.08–50.45)	26.76 (19.72–50.61)	0.738
Race				0.187			0.141
	White	29 (85.29%)	59 (83.10%)		22 (81.48%)	56 (83.58%)	
	Asian	4 (11.76%)	5 (7.04%)		4 (14.81%)	4 (5.97%)	
	Black or African American	0	6 (8.45%)		0	6 (8.96%)	
Masaoka stage				0.651			0.74
	I	9 (26.47%)	26 (36.62%)		8 (29.63%)	24 (35.82%%)	
	IIa and IIb	20 (58.52%)	35 (49.30%)		16 (59.26%)	35 (52.24%%)	
	III	4 (11.76%)	8 (11.27%)		2 (7.41%)	7 (10.45%)	
	IVa and IVb	1 (2.94)	1 (1.41%)		1 (3.70%)	1 (1.49%)	
Histological type				0.025^*^			0.261
	Type A	3 (8.82%)	12 (16.90%)		3 (11.11%)	12 (17.91%)	
	Type AB	8 (23.53%)	26 (36.62%)		8 (29.63%)	24 (35.82%)	
	Type B1	2 (5.88%)	13 (18.31%)		2 (7.41%)	12 (17.91%)	
	Type B2	15 (44.12%)	15 (21.13%)		12 (44.44%)	15 (22.39%)	
	Type B3	6 (17.65%)	5 (7.04%)		2 (7.41%)	4 (5.97%)	

*BMI, body mass index*;

* *p < 0.05*.

### Differential Expression Analysis and Enrichment Analysis

**Figure 1:**
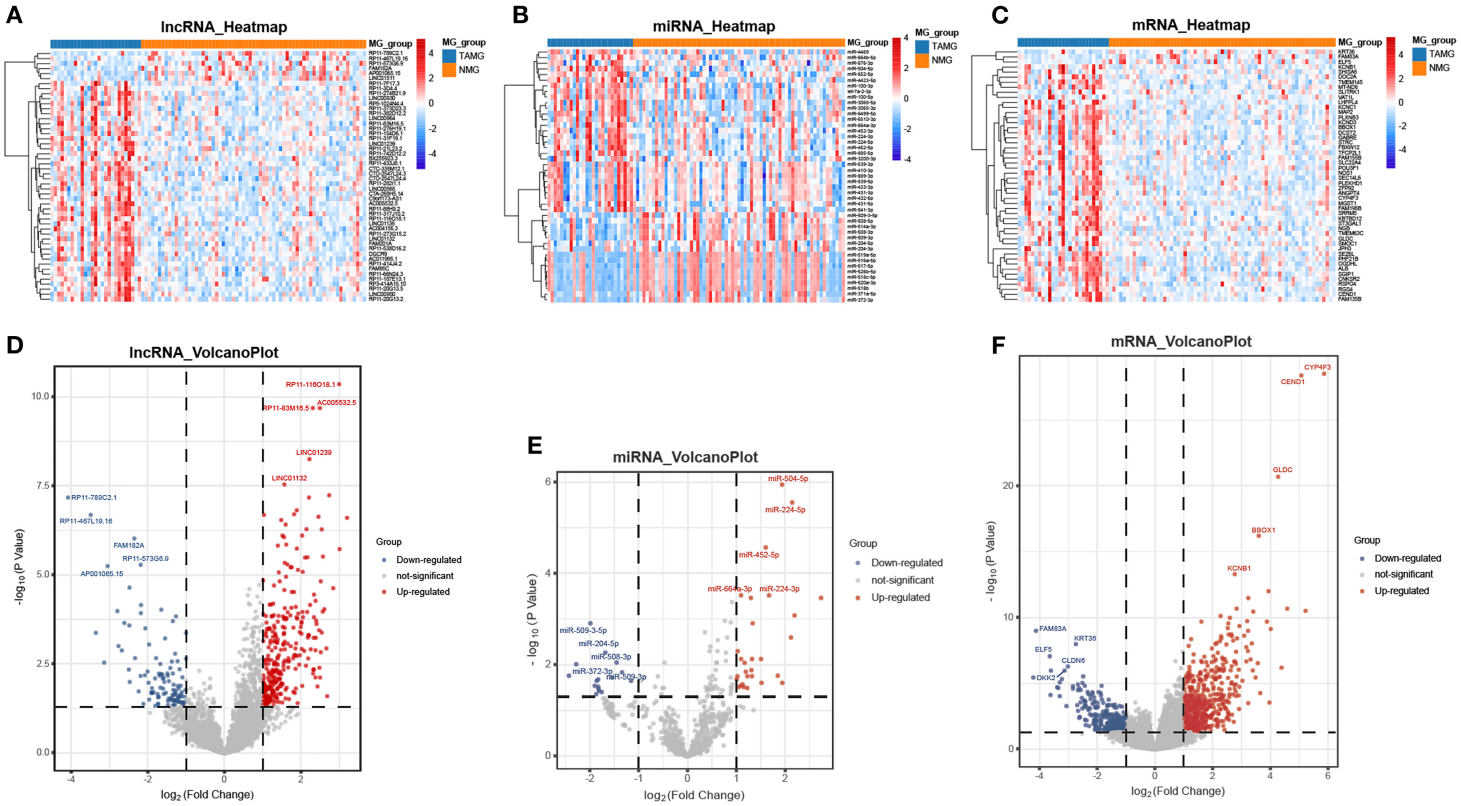
Differentially expressed genes between TAMG and NMG. **(A)** Heatmap of the 50 most differentially expressed lncRNAs; **(B)** Heatmap of the 45 differentially expressed miRNAs; **(C)** Heatmap of the 50 most differentially expressed mRNAs; **(D–F)** Volcano plot of differentially expressed lncRNAs, miRNAs, and mRNAs. The five most upregulated and five most downregulated genes are marked in the volcano plots.

We performed differential expression analysis based on the RNA-seq data of 94 patients. A total of 847 DEmRNAs (559 upregulated, 288 downregulated), 409 DElncRNAs (300 upregulated, 109 downregulated), and 45 DEmiRNAs (29 upregulated, 16 downregulated) were identified (Figures 1A–F).

**Figure 2:**
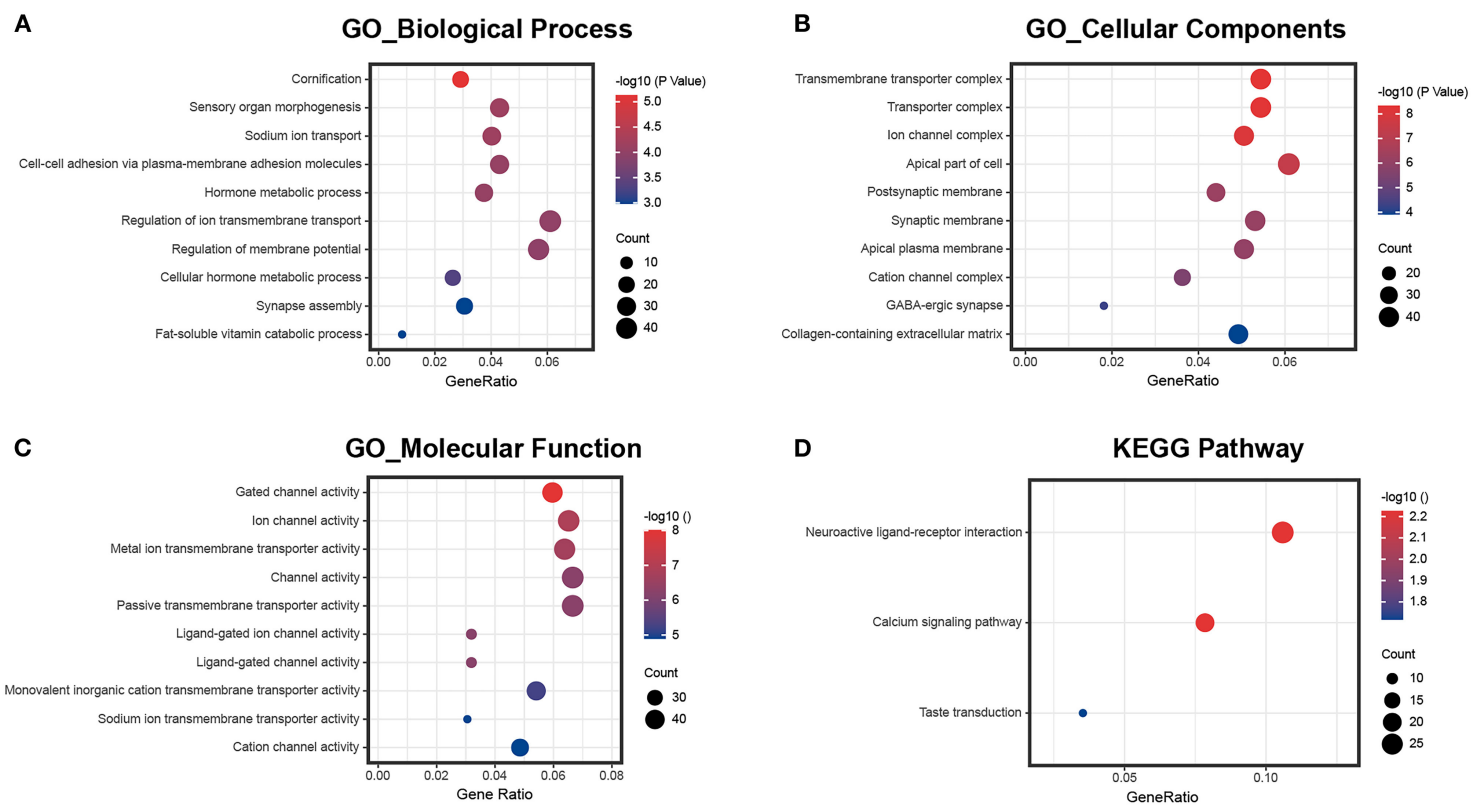
GO and KEGG pathway enrichment of differentially expressed mRNAs. **(A)** The ten most enriched biological process GO terms; **(B)** the ten most enriched cellular component GO terms; **(C)** the ten most enriched molecular function GO terms; **(D)** KEGG pathway enrichment analysis.

Then, according to the DEmRNAs, we conducted the GO and KEGG enrichment analyses. As shown in Figures 2A–D, the enriched GO terms in these biological process (BP) were regulators of ion transmembrane transport (gene ratio = 6.10%, p.adj < 0.001), regulation of membrane potential (gene ratio = 5.69%, p.adj < 0.001), sensory organ morphogenesis (gene ratio = 4.30%, p.adj = 7.37 × 10^−5^), cell–cell adhesion *via* plasma-membrane adhesion molecules (gene ratio = 4.30%, p.adj = 9.34 × 10^−5^), and sodium ion transport (gene ratio = 4.02%, p.adj = 7.37 × 10^−5^). The enriched GO terms in cellular components (CC) were apical part of cell (gene ratio = 6.52%, p.adj = 4.20 × 10^−8^), transmembrane transporter complex (gene ratio = 5.83%, p.adj = 4.61 × 10^−9^), transporter complex (gene ratio = 5.83%, p.adj = 5.14 × 10^−9^), synaptic membrane (gene ratio = 5.69%, p.adj = 7.68 × 10^−7^), and ion channel complex (gene ratio = 5.41%, p.adj = 8.54 × 10^−9^). The enriched GO terms in molecular function (MF) were channel activity (gene ratio = 6.66%, p.adj = 5.96 × 10^−7^), passive transmembrane transporter activity (gene ratio = 6.66%, p.adj = 5.96 × 10^−7^), ion channel activity (gene ratio = 6.52%, p.adj = 1.28 × 10^−7^), metal ion transmembrane transporter activity (gene ratio = 6.38%, p.adj = 2.13 × 10^−7^), and gated channel activity (gene ratio = 5.96%, p.adj = 9.66 × 10^−9^). The KEGG pathways were enriched in neuroactive ligand–receptor interaction (gene ratio = 10.59%, p.adj = 0.006), calcium signaling pathway (gene ratio = 7.84%, p.adj = 0.006), and taste transduction (gene ratio = 3.53%, p.adj = 0.019).

### Construction of the ceRNA Network

**Figure 3:**
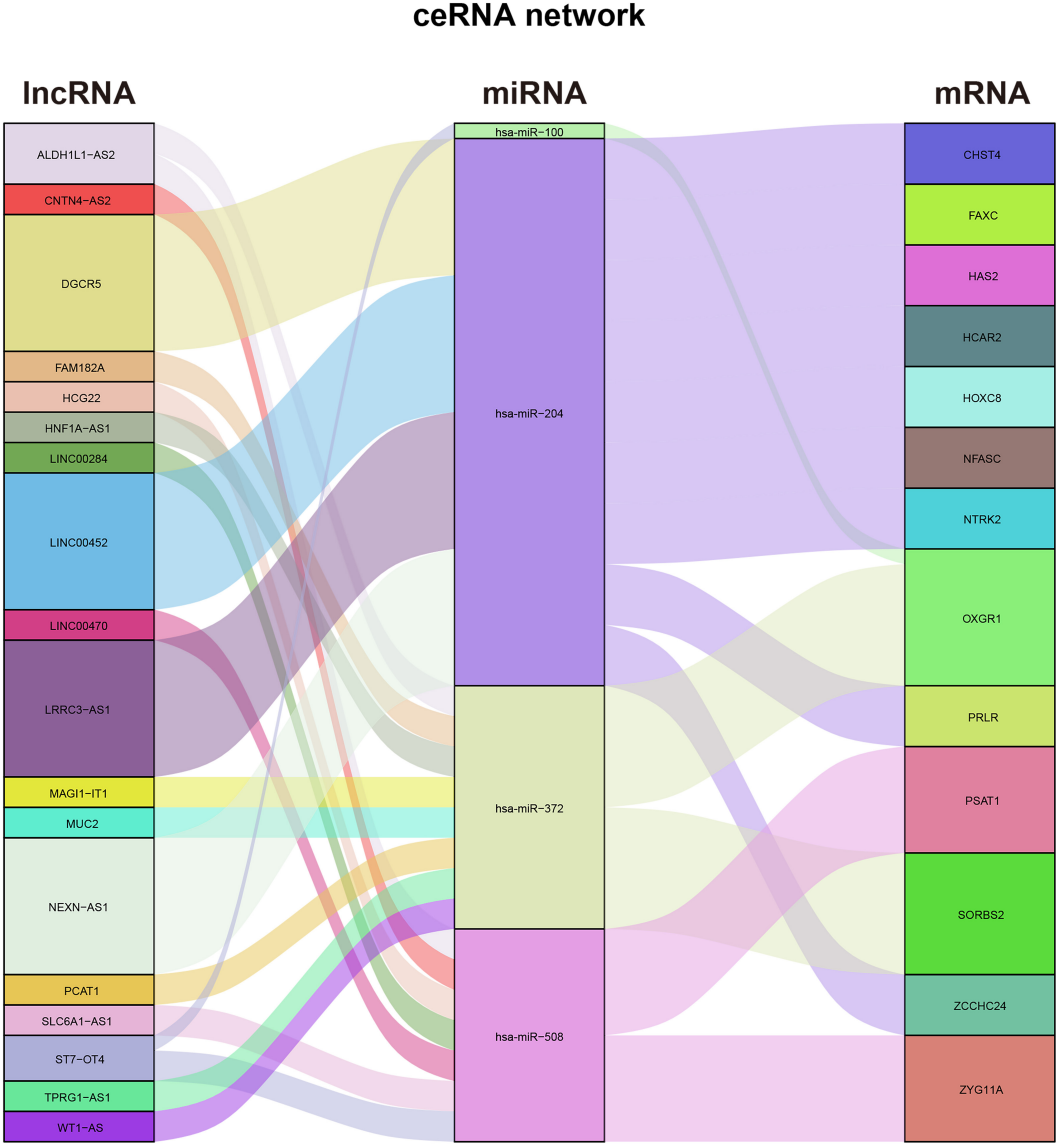
Alluvial plot of the ceRNA network in patients with TAMG.

First, we predicted 36 DEmiRNA–DElncRNA pairs, including six DEmiRNAs and 18 DElncRNAs, based on miRcode. Then, based on the DEmRNAs and these six predicted DEmiRNAs, we predicted 14 DEmiRNA–DEmRNA pairs, including four DEmiRNAs and 13 DEmRNAs, utilizing the TargetScan, miRTarBase, and miRDB databases. Finally, we merged the predicted DEmiRNA–DElncRNA pairs and DEmiRNA–DEmRNA pairs and successfully constructed the ceRNA network of differentially expressed RNAs in the thymoma of TAMG (Figure 3).

### Immune Features of Thymoma in TAMG

**Figure 4:**
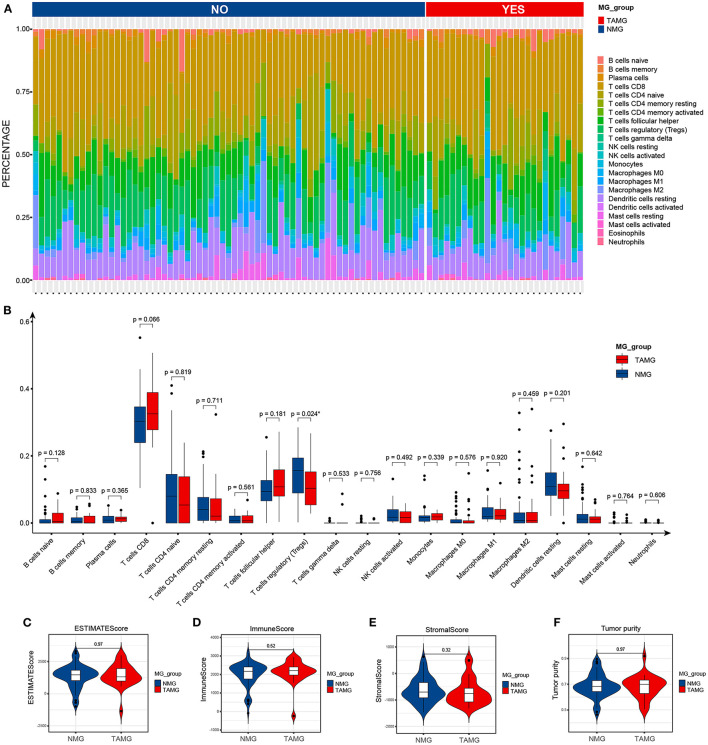
Immune features of thymoma in patients with TAMG. **(A)** Bar plots show the composition of immune cells predicted by using the CIBERSORT algorithm in thymoma of TAMG and patients with NMG. **(B)** Box plots denote the estimated proportion of immune cells in thymoma between patients with TAMG and NMG. Violin plots demonstrate the ESTIMATE score **(C)**, immune score **(D)**, stromal score **(E)** and tumor purity **(F)** calculated by use of the ESTIMATE algorithm.

By using the ESTIMATE algorithm, we calculated the stromal score, immune score, ESTIMATE score, and tumor purity to assess the overall patterns of the immune microenvironment of thymoma; however, there was no difference in the overall patterns of the immune microenvironment of thymoma between TAMG and NMG (Figures 4C–F). We then compared the proportions of TIICs calculated by using the CIBERSORT algorithm between the groups (Figure 4A). According to the Wilcoxon test, the proportion of regulatory T cells (Tregs) in the TAMG was significantly lower than that in the NMG (*p* = 0.024, Figure 4B).

### Possible Correlation Between the ceRNA Network and Tregs in Thymoma

**Figure 5:**
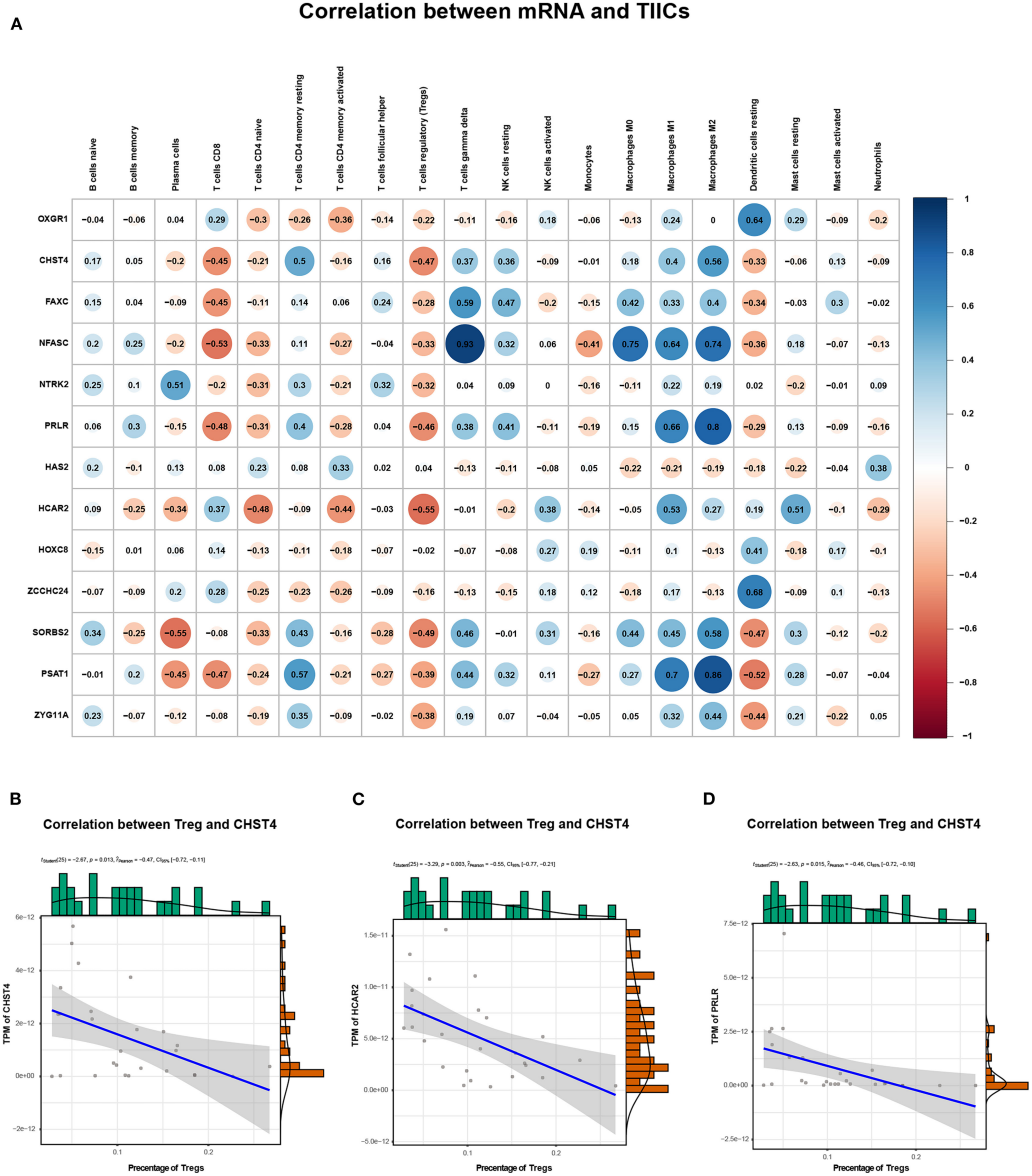
Correlation between mRNAs in the ceRNA network and estimated immune cells. **(A)** shows the Pearson correlation coefficient between mRNAs in the ceRNA network and immune cells. **(B)** illustrates the correlation between CHST4 and Tregs (*r* = −0.47, *p* = 1.312 × 10^−2^); **(C)** shows the correlation between HCAR2 and Tregs (*r* = −0.55, *p* = 2.956 × 10^−3^); **(D)** shows the correlation between PRLR and Tregs (*r* = −0.47, *p* = 1.457 × 10^−2^). In **(B–D)**, the blue line represents a linear mode indicating the relationship between mRNA expression (TPM value) and the percentage of Tregs (the gray area represents the 95% confidence interval).

**Figure 6:**
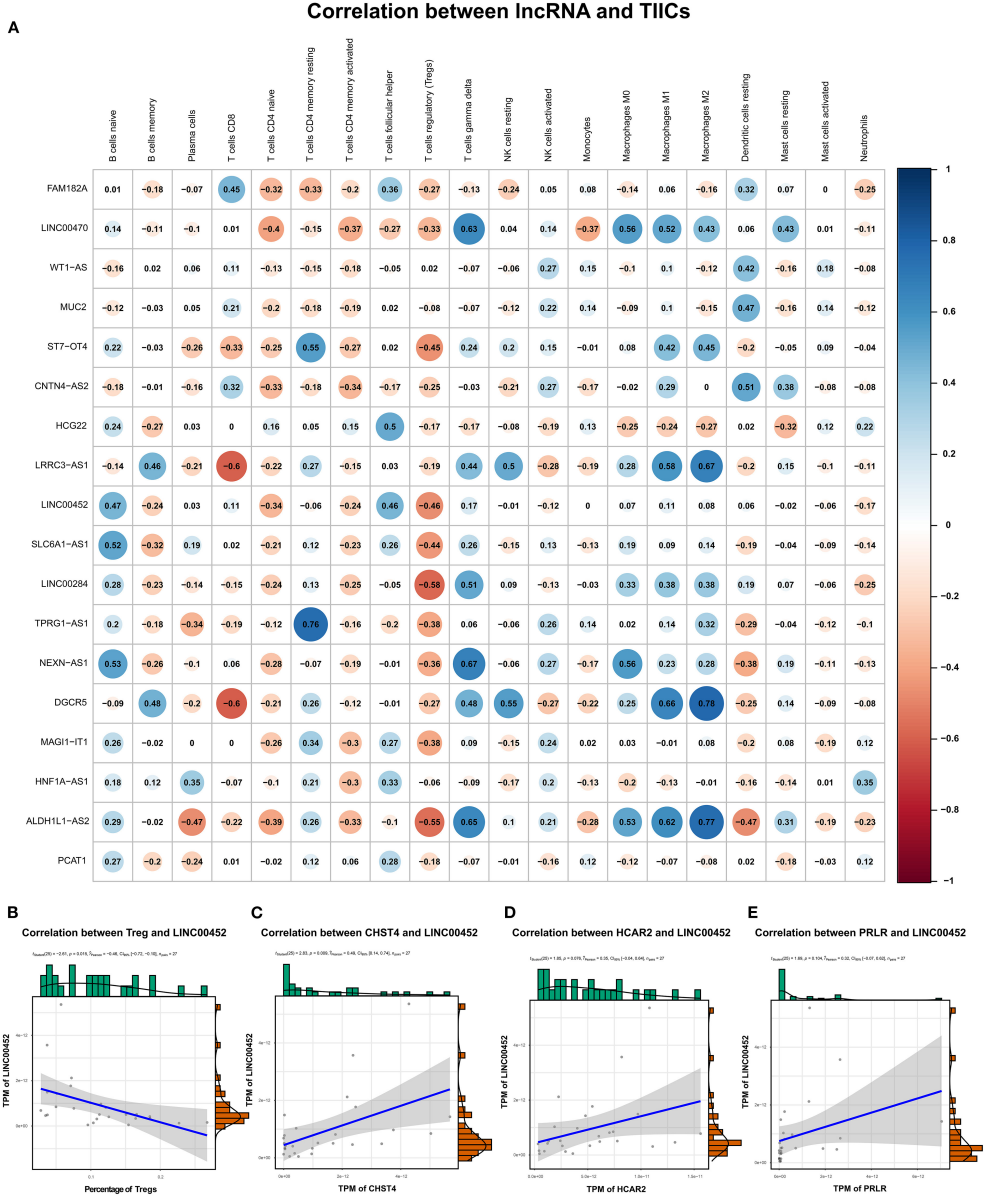
Correlation between lncRNAs in the ceRNA network and estimated immune cells. **(A)** shows the Pearson correlation coefficient between lncRNAs in the ceRNA network and immune cells. **(B)** demonstrates the correlation between LINC00452 and Tregs (*r* = −0.46, *p* = 0.015); **(C)** shows the correlation between LINC00452 and CHST4 (*r* = 0.49, *p* = 8.953 × 10^−3^); **(D)** denotes the correlation between LINC00452 and HCAR2 (*r* = 0.35, *p* = 0.076); **(E)** shows the correlation between LINC00452 and PRLR (*r* = 0.32, *p* = 0.104). In **(B)**, the blue line denotes a linear mode indicating the relation between mRNA expression (TPM value) and the percentage of Tregs. In **(C–E)**, the blue line denotes a linear mode indicating the relationship between mRNA and lncRNA expression (TPM value). The gray area represents the 95% confidence interval.

**Figure 7:**
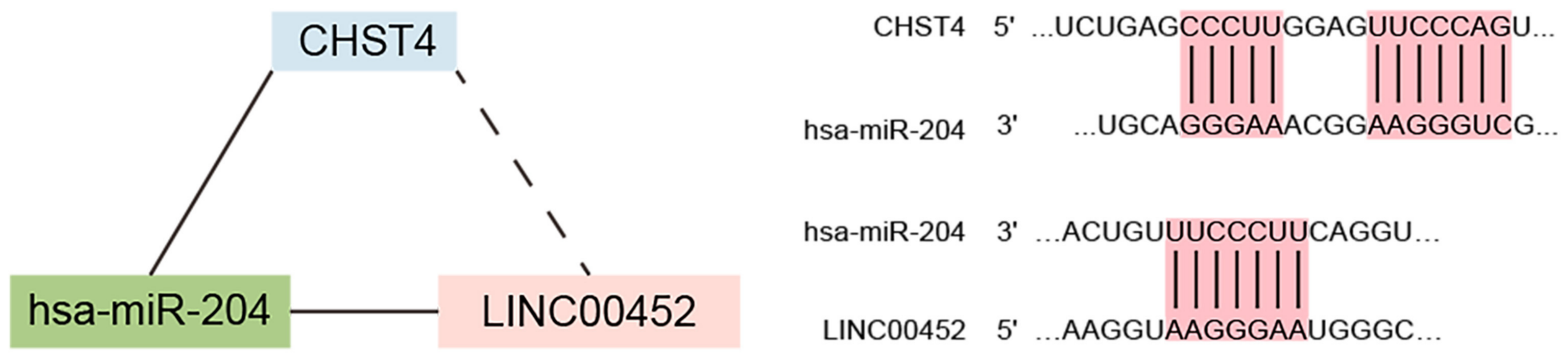
The crosstalk between CHST4, hsa-miR-204 and LINC00452 in patients with TAMG.

We explored the correlation between the predicted proportions of TIICs and RNAs in the ceRNA network by using the Pearson correlation method. As illustrated in Figure 5, for DEmRNAs, HCAR2 (*r* = −0.55, *p* = 2.956 × 10^−3^), SORBS2 (*r* = −0.49, *p* = 1.009 × 10^−2^), CHST4 (*r* = −0.47, *p* = 1.312 × 10^−2^), PRLR (*r* = −0.47, *p* = 1.457 × 10^−2^), PSAT1 (*r* = −0.39, *p* = 4.22 × 10^−2^), and ZYG11A (*r* = −0.39, *p* = 4.747 × 10^−2^) were significantly correlated with Tregs. Among them, the differences in HCAR2 (log_2_ FC = −2.17), CHST4 (log_2_ FC = 2.38), and PRLR (log_2_ FC = 2.34) between TAMG and NMG were more pronounced. Furthermore, for DElncRNAs, SLC6A1–AS1 (*r* = −0.44, *p* = 2.172 × 10^−2^), ALDH1L1–AS2 (*r* = −0.55, *p* = 2.747 × 10^−3^), ST7–OT4 (*r* = −0.45, *p* = 1.822 × 10^−2^), LINC00452 (*r* = −0.46, *p* = 1.517 × 10^−2^), LINC00284 (*r* = −0.58, *p* = 1.657 × 10^−3^), TPRG1–AS1 (*r* = −0.38, *p* = 4.862 × 10^−2^), and MAGI1–IT1 (*r* = −0.38, *p* = 4.986 × 10^−2^) were significantly correlated with Tregs (Figure 6). Based on the ceRNA network we built, LINC00452 might regulate the expression of HCAR2, CHST4, and PRLR by competitively binding miR-204. We further assessed the correlation between LINC00452 and three mRNAs (HCAR2, CHST4, and PRLR). The results of Pearson correlation analysis showed that only LINC00452 was significantly and positively correlated with CHST4 (*r* = 0.49, *p* = 8.953 × 10^−3^) in the thymoma of patients with TAMG (Figures 6C–E, 7).

### Functional Prediction of CHST4 in Patients With Thymoma With MG

**Figure 8:**
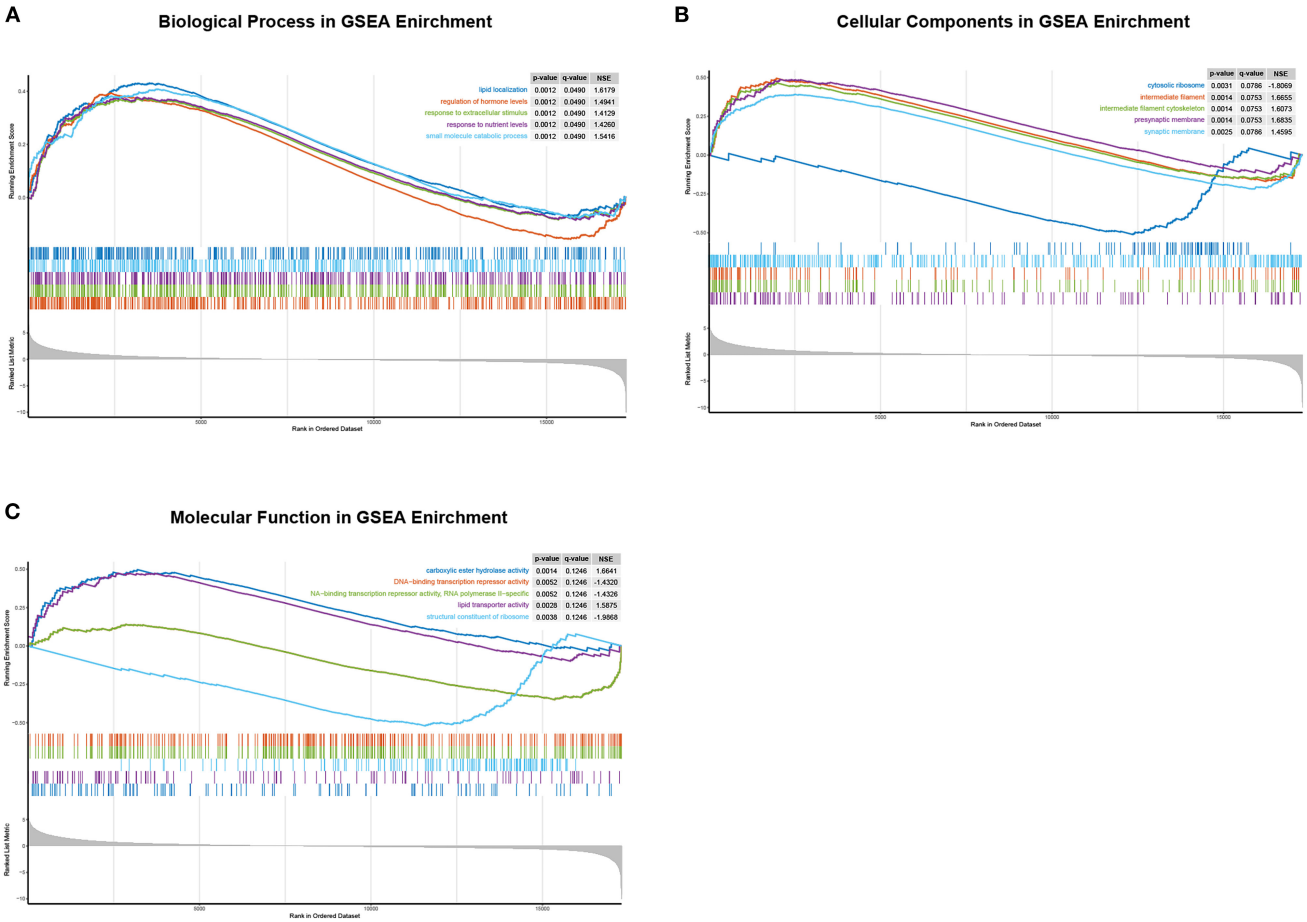
GO enrichment analysis of differentially expressed genes between the high- and low-CHST4 expression groups in TAMG patients using GSEA. **(A)** The biological process in GSEA; **(B)** The cellular components in GSEA; **(C)** The molecular function in GSEA; In **(C)**, the curve representing “DNA-binding transcription repressor activity” was covered by that representing “DNA-binding transcription repressor activity, RNA polymerase II-specific”; these two curves are of similar shape.

To further analyze the function of CHST4 in TAMG, we divided the patients with TAMG into high- and low-CHST4 expression groups according to the median TPM of CHST4. Differential gene expression analysis, GO functional enrichment, and KEGG pathway enrichment were then conducted. The results of differential gene expression analysis are shown in Figure 8.

According to the results of functional enrichment analysis by GSEA, for the BP category, the high-expression group was mainly enriched in the regulation of hormone levels [normalized enrichment scores (NES) = 1.49, *p* = 0.001, *q* = 0.049], response to extracellular stimulus (NES = 1.41, *p* = 0.001, *q* = 0.049), response to nutrient levels (NES = 1.43, *p* = 0.001, *q* = 0.049), small-molecule catabolic processing (NES = 1.54, *p* = 0.001, *q* = 0.049), and lipid localization (NES = 1.62, *p* = 0.001, *q* = 0.049). For the CC category, presynaptic membrane (NES = 1.68, *p* = 0.001, *q* = 0.075), intermediate filament cytoskeleton (NES = 1.61, *p* = 0.001, q = 0.075), intermediate filament (NES = 1.67, *p* = 0.001, *q* = 0.075), synaptic membrane (NES = 1.46, *p* = 0.002, *q* = 0.079), and cytosolic ribosome (NES = −1.81, *p* = 0.003, *q* = 0.079) were mainly enriched. For the MF category, carboxylic ester hydrolase activity (NES = 1.66, *p* = 0.001, *q* = 0.125), lipid transporter activity (NES = 1.59, *p* = 0.003, *q* = 0.125), structural constituent of ribosome (NES = −1.99, *p* = 0.004, *q* = 0.125), DNA-binding transcription repressor activity, RNA polymerase II-specific (NES = −1.43, *p* = 0.005, *q* = 0.125), and DNA-binding transcription repressor activity (NES = −1.43, *p* = 0.005, *q* = 0.125) were mainly enriched; however, there was no pathway significantly enriched in the high-CHST4 expression group according to the KEGG pathway enrichment analysis.

### The Expression of Genes of Interest in Thymic Tissue

**Figure 9:**
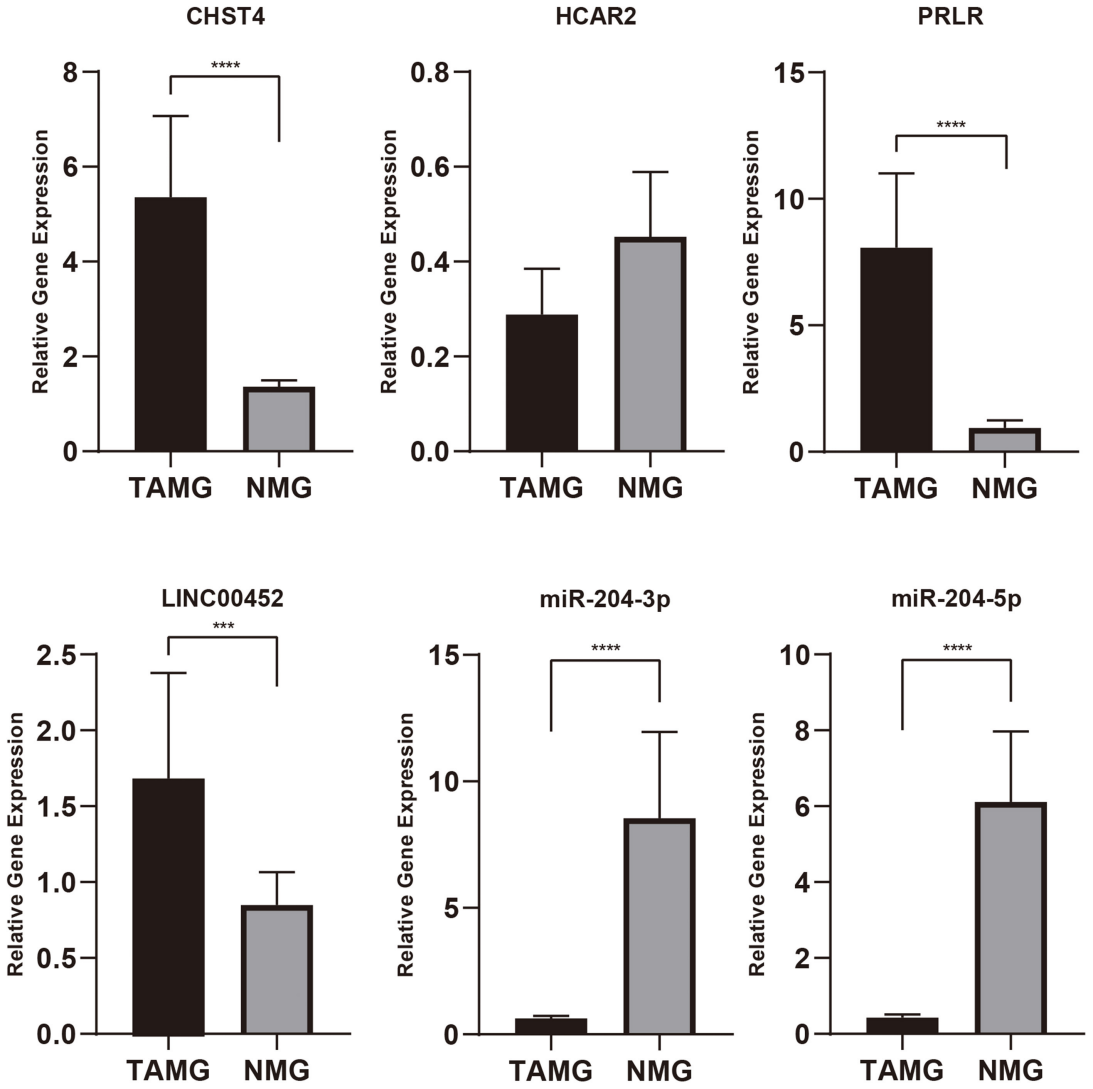
The relative expression of genes of interest in thymic tissue by qPCR. As shown in this figure, CHST4, PRLR, and LINC00452 were highly expressed in patients with TAMG, and miR-204-3p and miR-204-5p were expressed at low levels in patients with TAMG. n.s., not significant; ***p < 0.001; ****p < 0.0001.

The primer sequences are detailed in Table 2. As shown in Figure 9, CHST4, PRLR, and LINC00452 were highly expressed in the thymic tissue of TAMG, which is in accordance with previous results (*p* < 0.001). Moreover, miR-204-3p and miR-204-5p were expressed at low levels in the thymic tissue of patients with TAMG (*p* < 0.0001). However, HCAR2 was not differentially expressed between the groups (*p* = 0.3955).

**Table 2:** The primer for qPCR.

**Gene**	**Type**	**Sequence (5' → 3')**
CHST4	Forward	ACGCTTTCCACACAAATGCC
	Reverse	TCATAGGGCAAAGACCAGCG
PRLR	Forward	AAACTGGTTGGTTCACGCTC
	Reverse	ACAGAGATCCACACGGTTGT
HCAR2	Forward	GACAACTATGTGAGGCGTTGG
	Reverse	AATACCTGTCTACCGCCACC
LINC00452	Forward	ACCGTGTCTTTCCCGTGTAT
	Reverse	GCACTGGTCACTCACAAACC
ACTINB	Forward	CCTTCCTGGGCATGGAGTC
	Reverse	TGATCTTCATTGTGCTGGGTG
U6	Forward	CTCGCTTCGGCAGCACA
	Reverse	AACGCTTCACGAATTTGCGT
miR-204-3p	For reverse transcription	GTCGTATCCAGTGCAGGGTCCGAGGTATT CGCACTGGATACGACACGTCC
	Forward	CGGCTGGGAAGGCAAAG
	Reverse	AGTGCAGGGTCCGAGGTATT
miR-204-5p	For reverse transcription	GTCGTATCCAGTGCAGGGTCCGAGGTATTC GCACTGGATACGACAGGCAT
	Forward	CGCATTCCCTTTGTCATCCT
	Reverse	AGTGCAGGGTCCGAGGTATT

## Discussion

In this research, we studied the differentially expressed RNAs and their possible function between thymoma patients with and without MG, constructed a ceRNA network that might participate in the progression of TAMG, compared the patterns of the immune microenvironment and TIICs between thymoma patients with and without MG, and analyzed the function of CHST4 in thymoma patients with MG. We found that the differentially expressed mRNAs were mainly enriched in receptor-ligand activity, cytokine activity, and synaptic membrane, which was consistent with previous studies on MG. Moreover, despite the difference in the general patterns of the immune microenvironment in thymoma between groups being small, we found that the ratio of Treg cells in thymoma of patients with TAMG was larger than that in thymoma of patients with NMG. These findings confirmed that impaired Treg cells in thymoma play crucial roles in the progression of TAMG. Additionally, based on the resulting ceRNA network, the LINC00452/miR-204/CHST4 axis might regulate thymic Tregs, which may be involved in the development of TAMG. The findings of this research deepened our knowledge of the pathophysiology of TAMG.

The functional enrichment analysis suggested that differentially expressed genes were mainly enriched in receptor-ligand activity, synaptic membrane, channel activity, and transporter complex, which indicated that the nerve system in thymus might play an important role in the development of TAMG. Anatomically, the thymus was innervated by both sympathetic and parasympathetic nerve fibers (31). The neurotransmitter secreted by the nerve terminals can facilitate the maturation of thymocytes (31). Previous research has shown that the neuropeptide secreted by the nerve fibers in thymus, such as vasoactive intestinal polypeptide (VIP) and calcitoniny gene-related peptide (CGRP), shows important regulatory functions for immune system by regulating the differentiation, maturation, and apoptosis of thymocytes (31). Marie et al. (32) had shown that the receptor for VIP and CGRP might induce the expression of AChR α-chain and therefore might be involved in the development of MG. However, the neuropeptide is only one of the ways that the nervous system regulates the immune system, and whether other neuroregulatory mechanisms were involved in the development of MG requires further exploration. Notably, a research from Yamada et al. (33) has shown that the pathogenesis of TAMG might be quite different between the different thymoma histotypes. Therefore, further studies focusing on the pathogenesis of TAMG should consider the histotypes of thymoma.

The ceRNA hypothesis has been proven to be involved in the development and progression of various autoimmune diseases, such as MG, rheumatoid arthritis, and SLE (12, 13, 34). Previous research on the ceRNA network of MG showed that MALAT-1/miR-338-3p/MSL and SNHG16/let-7c-5p/IL-10 may participate in the regulation of MG (14, 15). Kong and colleagues found that the MALAT-1/miR-338-3p/MSL axis in peripheral blood mononuclear cells (PBMCs) may play a protective role in MG by inhibiting the activation of T cells, but the specific mechanism behind this inhibiting action needs further exploration (15). Furthermore, Wang and colleagues confirmed that SNHG16 may promote the expression of IL-10 by completely binding with let-7c-5p in PBMCs (14). Through this process, SNHG16 inhibits apoptosis and promotes apoptosis in Jurkat cells. Recently, Xu et al. (35) explored and discussed the gene methylation in the development of MG and predicted that LINC00173 might be involved in the progression of MG by regulating phosphatase and tensin homolog (PTEN). However, these studies mainly focused on the ceRNA network in peripheral blood, but the ceRNA network in the thymus that regulates MG is rarely discussed. In this study, we constructed a ceRNA network that may regulate the pathogenesis of MG in the thymoma of patients with TAMG, as evinced by data from several public databases, but the ceRNA network we constructed seems different from the network in previous studies. This might be attributed to the different mechanisms of MG in peripheral blood and in the thymus, as well as in thymoma and thymic hyperplasia (5, 36). Previous research showed that the characteristics of Treg cells differed between the thymus and the peripheral blood in patients with TAMG (37). Furthermore, CD4+ T helper (Th) cells mainly mediate the progression of TAMG, but B cells in ectopic germinal centers also play a crucial role in the progression of MG in patients with thymic hyperplasia (5). Our research mainly focuses on thymoma patients with and without MG, and the sequencing data used in this research were obtained from thymoma rather than from peripheral blood. Nevertheless, Wang and Kong mainly compared patients with MG and healthy people, and the sequencing data used in the two studies were obtained from PBMCs.

At the TIIC level, we found that the Treg cell counts in thymoma of patients with TAMG were significantly lower than those in thymoma of patients with NMG. Treg cells are immune cells that play very important roles in the maintenance of immune tolerance (38, 39). Physiologically, Treg cells suppress the function of conventional T (Tconv) cells to avoid excessive immune activation, which can induce autoimmune disease (40). In contrast to the normal thymus and hyperplastic thymus of patients with MG, the ratio of Treg cells was found to be significantly decreased in thymoma of patients with MG (41). Interestingly, the numbers of Treg cells in hyperplastic thymus and peripheral blood of patients with MG were comparable to those in thymus and peripheral blood of healthy people, but the suppression function of Treg cells was significantly impaired (42, 43). In summary, the changes in Treg cells are different in thymoma and hyperplastic thymus of patients with MG. This may be attributed to different mechanisms behind thymoma and thymic hyperplasia-related MG.

In has been explored that the pathology of tumor vessel is quite different between different thymoma subtypes (44). However, the tumor vasculature in TAMG is merely discussed (33). In this study, we found that the LINC00452/miR-204/CHST4 axis may regulate Treg cells in the progression of MG. CHST4 is expressed specifically in the high endothelial venule (HEV) (45). The *N*-acetylglucosamine 6-O sulfotransferase that CHST4 encoded is involved in the modification of l-selectin, which is crucial for lymphocyte homing (46–48). Previous research has shown that HEV plays a role in the formation of tertiary lymphoid organs (TLOs) in autoimmune disease by recruiting immune cells, which leads to epitope spreading and tissue injury therein (49). Through the HEVs in TLO, the immune cells from peripheral blood can be recruited to the thymus, resulting in thymic inflammation and leading to the development of autoimmune disease (50). Lefeuvre et al. had confirmed the existence of such a structure in thymic tissue around thymoma in patients with TAMG, which indicated that recruitment of peripheral immune cells through the HEV may promote the development of TAMG.

This research has certain shortcomings. First, the autoantibodies including, AChR-Ab and antibodies to muscle-specific tyrosine kinase (MuSK-Ab), are important when discussing the mechanism of MG. However, these data in the TCGA database are incomplete. Therefore, we cannot discuss the difference between the antibodies in this research. Second, we only analyzed the sequencing data from TCGA database but did not verify the results with data from the Gene Expression Omnibus (GEO) database due to the lack of RNA-seq data from TAMG and patients with thymoma in the GEO database. Third, experimental validations were required to confirm the findings of this research. Despite these limitations, this study adds to our understanding of RNA crosstalk in the regulation of thymic Tregs and the progression of thymoma-associated MG.

In conclusion, we constructed a ceRNA network with differentially expressed RNAs between thymoma patients with and without MG. Moreover, we found that the percentage of thymic Tregs was significantly decreased in patients with TAMG and demonstrated that the LINC00452/miR-204/CHST4 axis might regulate thymic Tregs in the progression of MG. These findings may deepen our understanding of the roles of the ceRNA network and thymic Treg cells in the development of TAMG and highlight the function of CHST4 in recruiting peripheral T cells in the progression of TAMG.

## Data Availability Statement

The datasets presented in this study can be found in online repositories. The names of the repository/repositories and accession number(s) can be found in the article/Supplementary Material.

## Ethics Statement

The studies involving human participants were reviewed and approved by Ethics Committee of West China Hospital of Sichuan University. The patients/participants provided their written informed consent to participate in this study.

## Author Contributions

YW, FW, and HZ designed the study, performed data interpretation, and participated in coordination. FW and HZ drafted the manuscript. GQ and ZL performed data collection and analysis. All authors have read and approved the final manuscript.

## Funding

This study was supported by the 1·3·5 project for disciplines of excellence-Clinical Research Incubation Project, West China Hospital, Sichuan University (2021HXFH056), the grants from the Key Research Project of Sichuan Province (No. 2020YFS0249), and National Key Research Project of China (No. 2017YFC0113502).

## Conflict of Interest

The authors declare that the research was conducted in the absence of any commercial or financial relationships that could be construed as a potential conflict of interest.

## Publisher's Note

All claims expressed in this article are solely those of the authors and do not necessarily represent those of their affiliated organizations, or those of the publisher, the editors and the reviewers. Any product that may be evaluated in this article, or claim that may be made by its manufacturer, is not guaranteed or endorsed by the publisher.
